# AmiA and AliA peptide ligands are secreted by *Klebsiella pneumoniae* and inhibit growth of *Streptococcus pneumoniae*

**DOI:** 10.1038/s41598-022-26838-z

**Published:** 2022-12-23

**Authors:** Janine Lux, Lalaina Holivololona, Raquel San Millan Gutierrez, Markus Hilty, Alban Ramette, Manfred Heller, Lucy J. Hathaway

**Affiliations:** 1 Institute for Infectious Diseases, Faculty of Medicine, University of Bern, Friedbühlstrasse 51, CH-3001 Bern, Switzerland; 2 Graduate School for Cellular and Biomedical Sciences, University of Bern, 3012 Bern, Switzerland; 3 Proteomics and Mass Spectrometry Core Facility, Department for BioMedical Research (DBMR), University of Bern, 3008 Bern, Switzerland

**Keywords:** Microbiology, Medical research

## Abstract

*Streptococcus pneumoniae* colonizes the human nasopharynx, a multi-species microbial niche. Pneumococcal Ami-AliA/AliB oligopeptide permease is an ABC transporter involved in environmental sensing with peptides AKTIKITQTR, FNEMQPIVDRQ, and AIQSEKARKHN identified as ligands of its substrate binding proteins AmiA, AliA, and AliB, respectively. These sequences match ribosomal proteins of multiple bacterial species, including *Klebsiella pneumoniae*. By mass spectrometry, we identified such peptides in the *Klebsiella pneumoniae* secretome. AmiA and AliA peptide ligands suppressed pneumococcal growth, but the effect was dependent on peptide length. Growth was suppressed for diverse pneumococci, including antibiotic-resistant strains, but not other bacterial species tested, with the exception of *Streptococcus pseudopneumoniae*, whose growth was suppressed by the AmiA peptide ligand. By multiple sequence alignments and protein and peptide binding site predictions, for AmiA we have identified the location of an amino acid in the putative binding site whose mutation appears to result in loss of response to the peptide. Our results indicate that pneumococci sense the presence of *Klebsiella pneumoniae* peptides in the environment.

## Introduction

The World Health Organization named *Streptococcus pneumoniae* (pneumococcus) as a priority pathogen in 2017^1^. These Gram-positive bacteria can cause pneumonia, septicemia, meningitis and otitis media in humans. This causes significant morbidity and mortality annually, especially in young children, the elderly and immunocompromised^2^. Current vaccines only induce immunity to a fraction of pneumococcal serotypes and do not protect against non-vaccine serotypes or nonencapsulated *S. pneumoniae*. This has led to a rise in the prevalence of antibiotic-resistant non-vaccine serotypes, making the search for new therapeutic agents imperative^3^.

Pneumococcus is usually an asymptomatic colonizer of the human nasopharynx, a niche which it shares with many other microorganisms and with which it must interact. *Klebsiella pneumoniae*, a Gram-negative bacterium, was first described following isolation from the lungs of a pneumonia patient in 1882^4^. *Klebsiella* are found in nature, e.g. in water, soil and animals^5^ and also colonize medical devices causing nosocomial infections^6^. As an opportunistic pathogen, *K. pneumoniae* colonizes mucosal surfaces, but may disseminate to other tissues causing urinary tract infections, pneumonia, bloodstream infections and sepsis^7^. A study in Semarang, Indonesia, found nasopharyngeal colonization of *K. pneumoniae* in 7% of healthy children (6–60 months old) and 15% of adults (45–70 years old)^8^*.* Gram-negative bacteria generally use acyl-homoserine lactones (auto-inducer 1) for communication, whilst Gram-positive bacteria use peptide-based molecules: autoinducing peptides (AIP) or quorum sensing peptides. Both Gram-positive and Gram-negative bacteria use auto-inducer 2 (AI2) for interspecies signaling^9,10^. In addition, we previously proposed that pneumococcus can detect bacteria within the same microbiota via short peptide fragments derived from other bacterial species that bind to the Ami-AliA/AliB permease^11–14^. Pneumococcal Ami-AliA/AliB oligopeptide permease is an ABC transporter involved in oligopeptide uptake, which is hypothesized to play a role in sensing changes in environmental conditions leading to modulation of gene expression^15^ and playing a role in nasopharyngeal colonization^16^. We previously identified peptides AKTIKITQTR, FNEMQPIVDRQ, and AIQSEKARKHN as ligands of its substrate binding proteins AmiA, AliA, and AliB, respectively^12^. These sequences match ribosomal proteins of multiple bacterial species, including those of the class of Gammaproteobacteria, which includes *Klebsiella* species^12^. The peptide ligands of AmiA, AliA and AliB proteins determine pneumococcal phenotypes, such as pneumococcal growth, biofilm production, capsule size, chain length and transformation rate^13^. Of particular interest, the AmiA and AliA ligands inhibited growth of pneumococcal laboratory strain D39 in a previous study^13^. From binding studies and growth curves with knock out mutants of the substrate binding proteins we deduced that there is some cross-reactivity: AmiA protein binds AmiA peptide but also, to some extent, AliA peptide whereas AliA protein binds AliA peptide and not AmiA peptide^12,13^.

Here, we aimed to determine whether the AmiA, AliA and AliB peptide ligands are indeed secreted by *K. pneumoniae*. Since a new method to suppress pneumococcal growth could be a valuable tool, we tested whether the AmiA and AliA ligands inhibit the growth of diverse clinical pneumococcal strains and whether the inhibitory effect is specific to *S. pneumoniae* or affects other bacterial species found in the nasopharyngeal microbiota. We predicted the AmiA and AliA protein structures and peptide binding sites. We propose that our study provides further insight into this novel route of interspecies bacterial communication and its potential in future therapeutics.

## Results

### *S. pneumoniae* growth is reduced by co-culture with *K. pneumoniae*

**Figure 1:**
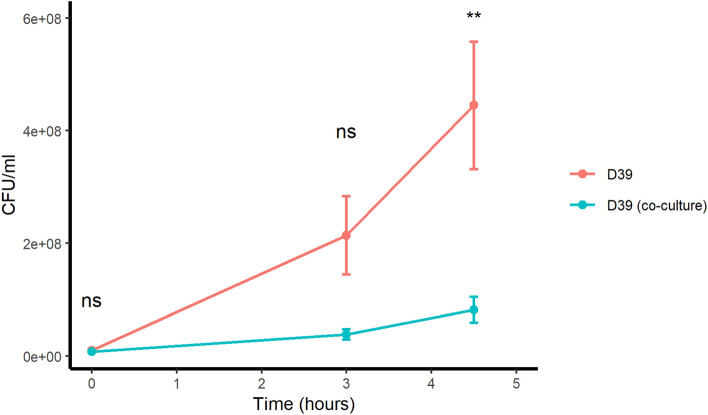
Growth of *S. pneumoniae* strain D39 in monoculture or co-culture with *K. pneumoniae ATCC BAA-1706* showing that by 4.5 h co-culture with *K. pneumoniae* had significantly reduced the growth of *S. pneumoniae* strain D39 compared to D39 growth in monoculture. Results represent the mean of 6 independent experiments, error bars indicate SEM, ** indicates *p* value < 0.01, ns indicates not significant by Wilcoxon test.

We cultured *S. pneumoniae* strain D39 in monoculture and in co-culture with *K. pneumoniae ATCC BAA-1706* in peptide-free chemically defined culture medium (CDM). By 4.5 h *S. pneumoniae* had grown significantly less in co-culture than in monoculture (Fig. 1), indicating that *K. pneumoniae* had reduced the growth of *S. pneumoniae*.

### *Klebsiella pneumoniae* secretes AmiA, AliA and AliB peptide ligands

We extracted peptides secreted by *K. pneumoniae* into CDM. To confirm that peptides were derived from live bacteria, we stained the *K. pneumoniae* and observed by fluorescent microscopy, which showed that more than 98% of the bacteria were alive (Supplementary Fig. S1). Peptides were extracted by solid-phase extraction and identified by LC-MS/MS. We used BLAST to search for similarity to AmiA, AliA and AliB ligands AKTIKITQTR, FNEMQPIVDRQ and AIQSEKARKHN respectively in the peptide pool. Two sequences in the *K. pneumoniae* secretome (peptides 1, 2 in Table 1) matched the AmiA ligand AKTIKITQTR, with one of them missing only the last amino acid. We identified the AliA ligand FNEMQPIVDRQ, as well as shorter and longer versions of it (peptides 3-10 in Table 1). The sequence EMQPIVDRQ was included in 7 out of these 8 peptides and therefore seems to be a consensus sequence. Sequences 11 and 12 in Table 1 match 30S ribosomal protein S20, which we found by BLAST to match the AliB ligand AIQSEKARKHN but with the second amino acid isoleucine (I) replaced by valine (V). Thus, versions of all the three previously identified AmiA, AliA and AliB peptide ligands were secreted by *K. pneumoniae*.

**Table 1:** Peptides with sequences of AmiA, AliA, and AliB peptide ligands were identified in *K. pneumoniae* secretome.

Peptide	Amino acid sequence	Possible origin determined by BLAST analysis
AmiA ligand	**AKTIKITQTR**	50S ribosomal subunit protein L30 (multispecies, including *Klebsiella pneumoniae, Escherichia coli*, *Salmonella enterica*, *Pantoea agglomerans*, *Proteus mirabilis* and *Mycobacterium tuberculosis*)
1	AKTIKITQT	
2	TQTRSAIGRLPKHKA	
AliA ligand	**FNEMQPIVDRQ**	30S ribosomal protein S20 (multispecies, including *Klebsiella pneumoniae, Escherichia coli*, *Salmonella enterica*, *Citrobacter freudii*)
3	EMQPIVDRQ	
4	EMQPIVDRQA	
5	NEMQPIVDRQ	
6	FNEMQPIVDRQ	
7	FNEMQPIVDRQA	
8	FNEMQPIVDRQAA	
9	FNEMQPIVDRQAAKG	
10	IVDRQAAKGLIHKNK	
AliB ligand	**AIQSEKARKHN**	30S ribosomal protein S20 (multispecies, including *Klebsiella pneumoniae, Shigella flexneri*, *Escherichia coli, Escherichia albertii*)
11	ANIKSAKKRAVQSEKARKHN	
12	ANIKSAKKRAVQSEKARK	

Peptides secreted by *K. pneumoniae* were identified by LC–MS and their sequences compared to those of the AmiA, AliA and AliB peptide ligands AKTIKITQTR, FNEMQPIVDRQ, and AIQSEKARKHN respectively, using BLAST. These previously identified peptide sequences are shown in bold, peptides identified in the secretome of *K. pneumoniae* are numbered 1–12. Possible origins of the peptides as determined by BLAST are shown in the third column^12^.

### Only the 9–11 amino acid peptides inhibit growth of *S. pneumoniae*

**Figure 2:**
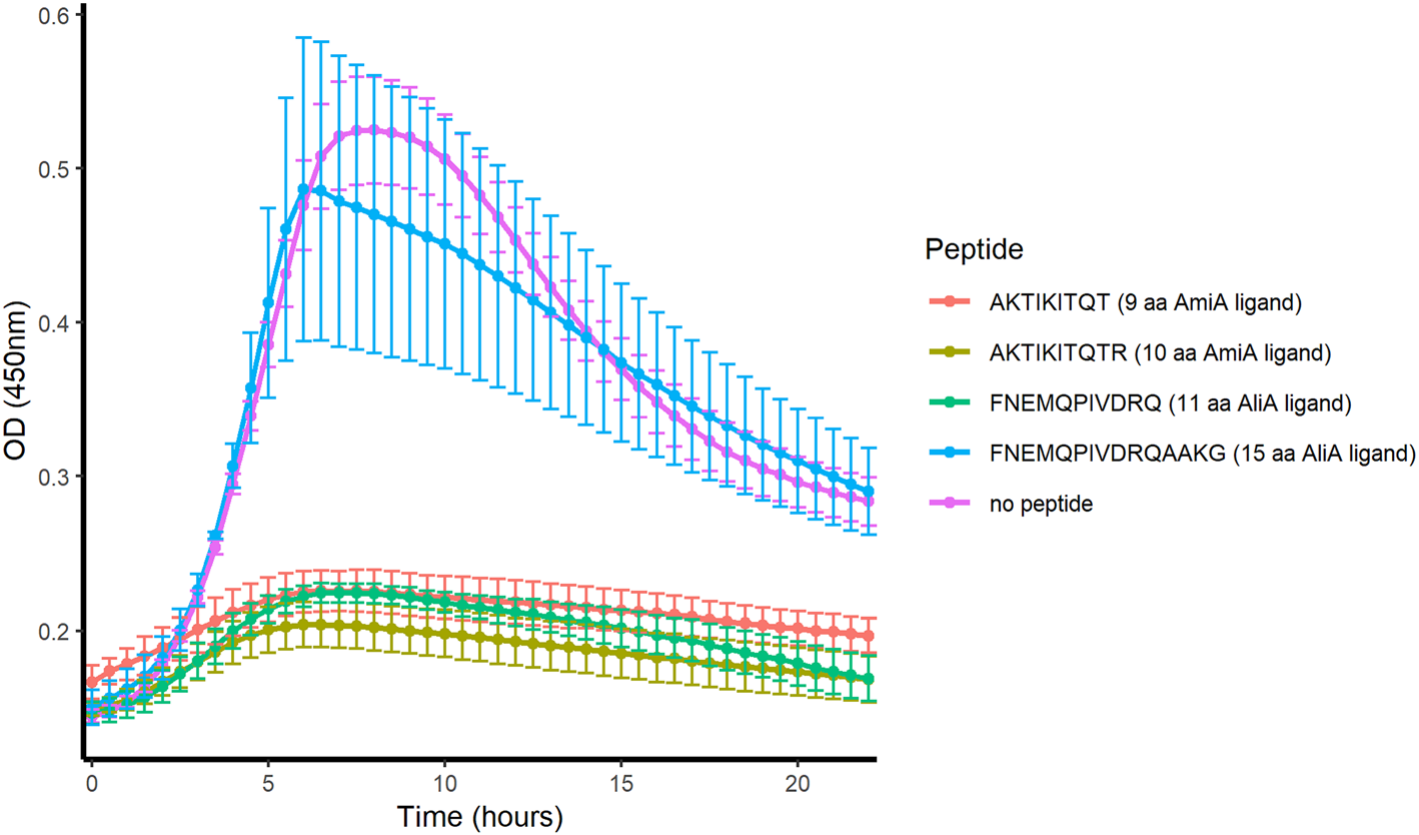
Growth curves of *S. pneumoniae* strain D39 in presence or absence of AmiA or AliA peptide ligands. Growth was monitored in the absence of peptides or the presence of 0.5 mg/ml AmiA peptide ligand AKTIKITQTR, AmiA peptide ligand lacking the final amino acid (peptide 1 in Table 1), AliA peptide ligand FNEMQPIVDRQ, and the longer (15 amino acids) version of AliA peptide ligand (peptide 9 in Table 1). Growth was measured in peptide-free chemically defined medium (CDM) by measuring optical density (OD) over time. Results represent 3 independent experiments, error bars indicate SEM.

We previously showed that AmiA peptide ligand AKTIKITQTR and AliA peptide ligand FNEMQPIVDRQ inhibit the growth of *S. pneumoniae* laboratory strain D39. We found different length versions of these peptides in the *K. pneumoniae* secretome and tested here whether the length of the peptide affected growth inhibition. We tested peptide 1 and peptide 9 from Table 1 together with the previously identified AmiA and AliA peptide ligands. We found that shorter, 9 amino acid, AmiA ligand version AKTIKITQT (peptide 1) inhibited growth of D39. However, the longer, 15 amino acid, version of AliA ligand, FNEMQPIVDRQAAKG (peptide 9), lost its growth-inhibiting effect (Fig. 2).

### Effect of AmiA and AliA peptide ligands on diverse pneumococcal isolates and other bacterial species of the nasopharynx

**Figure 3:**
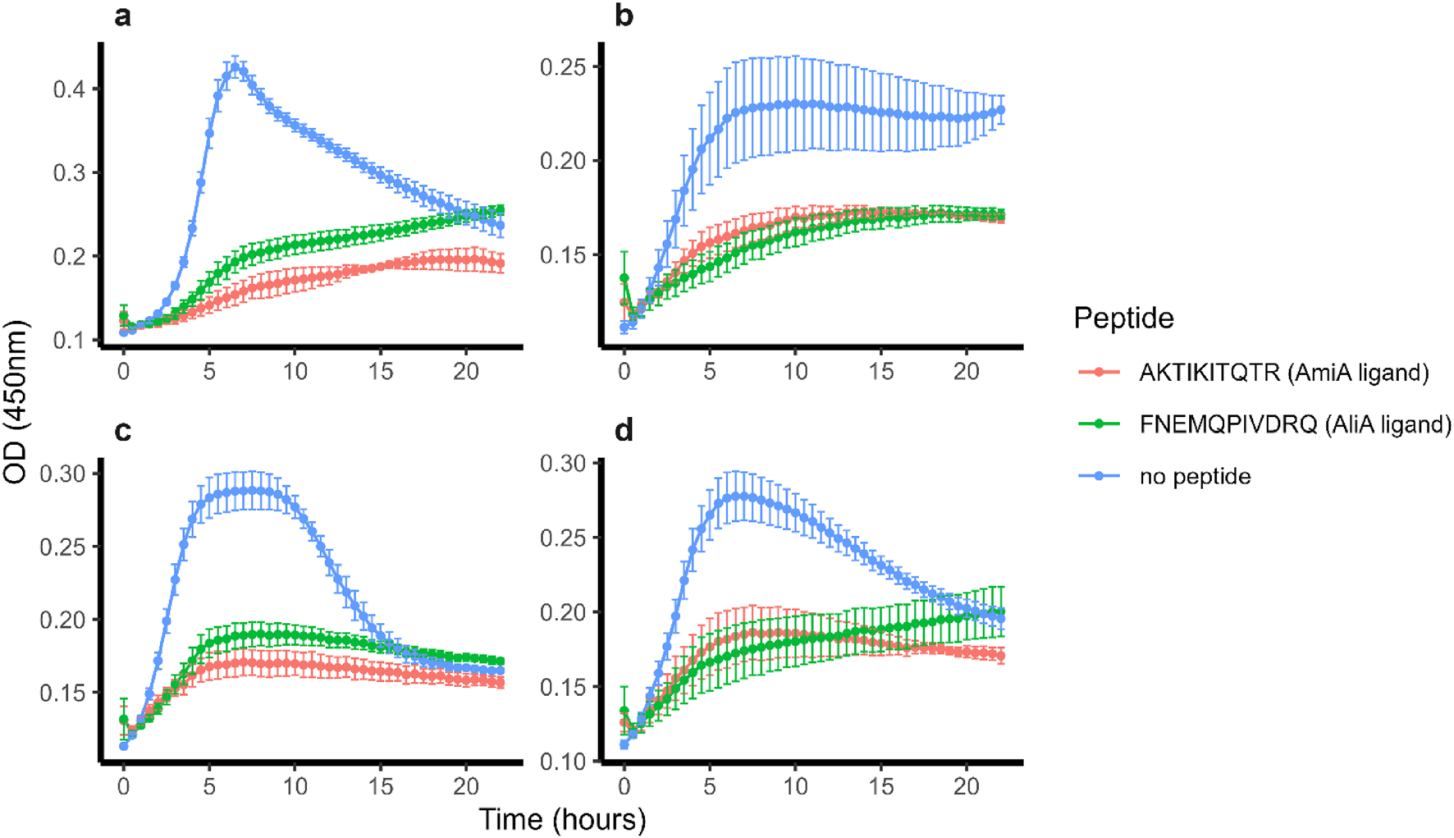
AmiA and AliA peptide ligand inhibited growth of Swiss clinical isolates of *S. pneumoniae* with strain numbers (**a**) 111.46, (**b**) 202.58, (**c**) 208.41 and (**d**) 307.17. Growth curves were measured in the absence of peptides or the presence of 0.5 mg/ml AmiA peptide ligand or AliA peptide ligand. Growth was measured in CDM by measuring optical density (OD) over time. Results represent 3 independent experiments, error bars indicate SEM.

**Figure 4:**
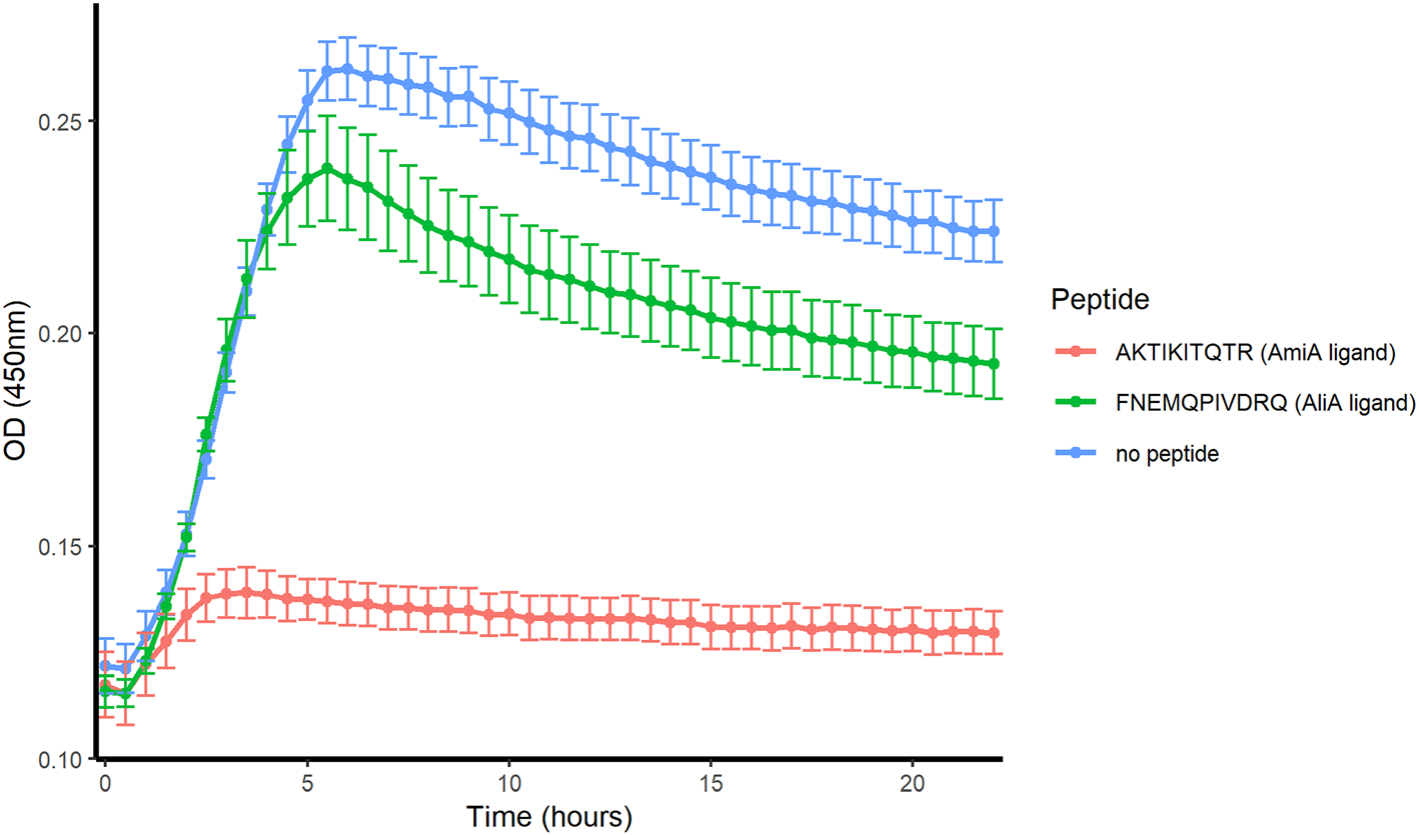
AmiA peptide ligand inhibited the growth of *S. pseudopneumoniae* strain 410.05, but AliA peptide ligand had little effect. Growth curves of clinical isolate of *S. pseudopneumoniae* (410.05) were measured in the absence of peptides or the presence of 0.5 mg/ml AmiA peptide ligand or AliA peptide ligand. Growth was measured in CDM by measuring optical density (OD) over time. Results represent 3 independent experiments, error bars indicate SEM.

BLAST analysis indicated that AmiA and AliA proteins are highly conserved between pneumococcal strains. The respective protein sequences of the D39 proteome are more than 99% identical with alignment scores ≥ 200 among the top 100 results, which represent sequences of diverse pneumococcal strains. Therefore, we tested whether AmiA and AliA peptide ligands were able to inhibit growth of genetically diverse clinical pneumococcal isolates, including antibiotic-resistant strains. For the Swiss clinical isolates tested, both AmiA and AliA peptides inhibited their growth (Fig. 3). The peptide concentrations inhibiting at least 50% of growth of all bacteria tested are shown in Table 2 with corresponding growth curves in Supplementary Fig. S2. Both peptides inhibited isolates of different serotypes and different genetic backgrounds, including a reference strain resistant to tetracycline and two clinical isolates with intermediate penicillin resistance. However, we found two *S. pneumoniae* strains, which were not inhibited by the AmiA and AliA peptide ligand: South African strain 19A-7 (ATCC 17619) and nonencapsulated strain R6. We tested whether the growth-inhibitory effect is species-specific by determining the effect of the peptides on other bacterial species of the nasopharyngeal microbiota. AmiA and AliA peptide ligands did not suppress growth of *Streptococcus mitis, Haemophilus influenzae, Staphylococcus aureus, Moraxella catarrhalis* or *Klebsiella pneumoniae*. AmiA peptide ligand inhibited the growth of *Streptococcus pseudopneumoniae*, but AliA peptide ligand had little effect (Fig. 4).

**Table 2:** MIC_50_ (minimum inhibitory concentration inhibiting at least 50% of bacteria growth) of AmiA and AliA peptide ligands on clinical Swiss *S. pneumoniae* strains, *S. pneumoniae* reference strains, including tetracycline-resistant strain ATCC 700903 and other species of the nasopharyngeal microbiota.

Species	Strain	Serotype	MLST	MIC_50_ AmiA ligand (mg/ml)	MIC_50_ AliA ligand (mg/ml)
*S. pneumoniae*	111.46 (Swiss)	19F	177	0.031	0.062
*S. pneumoniae*	202.58 (Swiss)	1	305	0.25	0.25
*S. pneumoniae*	208.41 (Swiss)	7F	191	0.125	0.125
*S. pneumoniae*	307.14 (Swiss)	18C	113	0.25	0.125
*S. pneumoniae*	ATCC 700903 (43362 Finland)	6B	270	0.5	0.25
*S. pneumoniae*	ATCC 17619 (South African 19A-7)	19A	75	X	X
*S. pneumoniae*	D39	2	595	0.031	0.5
*S. pneumoniae*	R6	Non typeable	595	X	X
*S. pseudopneumoniae*	410.05	*n.a*	*n.a*	0.062	X
*S. mitis*	3308.46	*n.a*	*n.a*	X	X
*H. influenzae*	ATCC 49247	*n.a*	*n.a*	X	X
*S. aureus*	ATCC 29213	*n.a*	*n.a*	X	X
*M. catarrhalis*	ATCC 8176	*n.a*	*n.a*	X	X
*K. pneumoniae*	ATCC BAA-1706	*n.a*	*n.a*	X	X

Strains 111.46 and 202.58 have intermediate resistance to penicillin. Lack of inhibition is indicated by "X" and not applicable by "*n.a.*".

### Comparison of AmiA and AliA between streptococcal species and pneumococcal isolates

**Figure 5:**
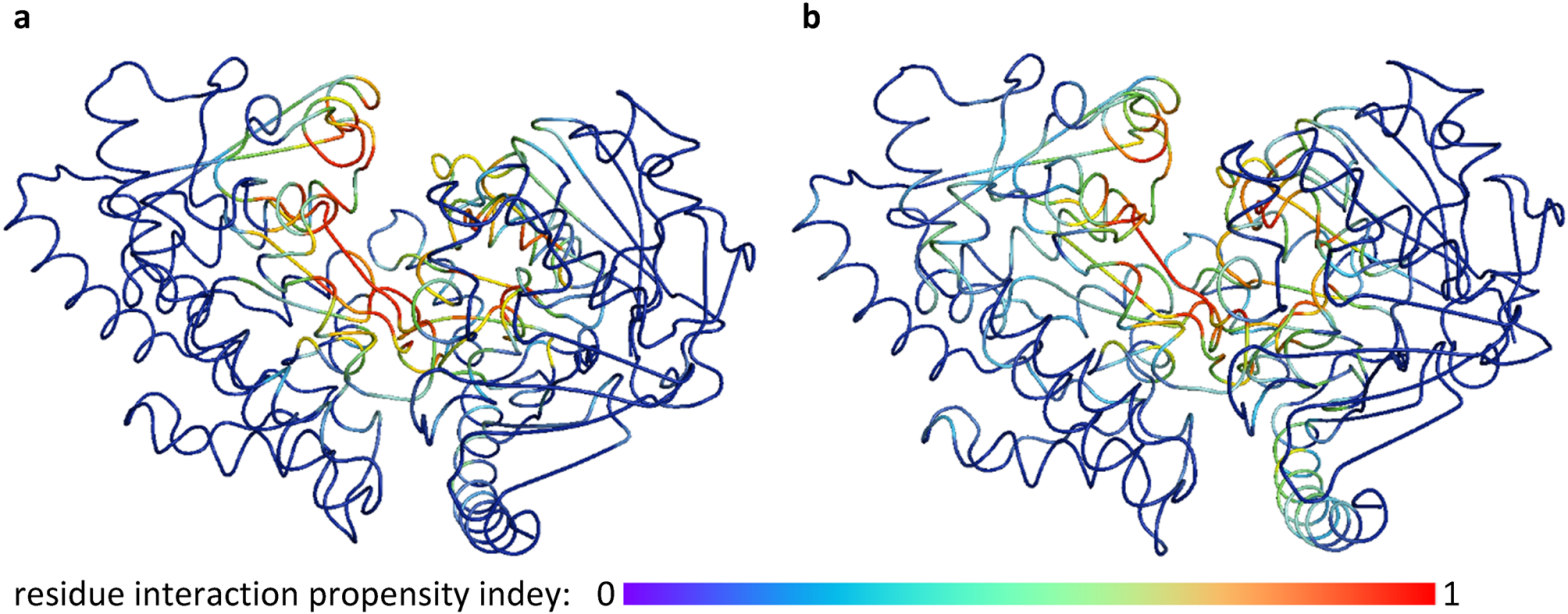
Prediction of peptide-protein interface of *S. pneumoniae* strain 110.58 for (**a**) AmiA and its peptide ligand (**b**) AliA and its peptide ligand. AlphaFold models of oligopeptide-binding proteins AmiA and AliA from 110.58 were used together with the sequence of AmiA and AliA peptide ligand respectively to predict the peptide-binding interface with PepNN. The colour represents the residue interaction propensity index, with 0 representing low index and low likelihood to be at peptide-protein interaction interface and 1 representing high index and likelihood to be at peptide-protein interaction interface. Visualization with PyMol.

To investigate why the growth of some isolates in Table 2 was inhibited by AmiA and AliA peptide ligands but others was not, we sequenced the genes encoding AmiA and AliA in the bacterial isolates used in the growth curves, predicted the translated product and compared the amino acid sequences. The Whole Genome Shotgun project has been deposited at DDBJ/ENA/GenBank under accessions JAMLJP000000000 (*S. pneumoniae* ATCC 17619), JAMLJQ000000000 (*S. pneumoniae* R6), and JAMLJR000000000 (*S. pneumoniae* D39). The percent homologies on nucleotide and amino acid level compared to strain D39 are shown in Table 3. As expected, compared to *S. pneumoniae* strain D39, strain R6 was the most homologous: sequence identity was 100% for AliA, but not AmiA (99.85%). AmiA and AliA of *S. pneumoniae* strain ATCC 17619 were also highly homologous (≥ 99% sequence identity) compared to strain D39, followed by the clinical isolates of *S. pseudopneumoniae* (89.55–98%) and *S. mitis* (77–86.97%)*.* Since the peptide ligands were originally identified by their binding to the AmiA and AliA proteins of strain 110.58^12^, we constructed 3D models of the proteins using the amino acid sequences without N-terminal signal peptide with AlphaFold^17,18^. We then used the protein structure files and the AmiA or AliA peptide ligand sequences to predict the localization of putative peptide binding sites. We did this by predicting the peptide-protein interaction interface with PepNN^19^, shown in Fig. 5. This information was used in the following sections to determine whether any amino acid difference between strains or species might affect binding sites.

**Table 3:** Percent homology (sequence identity) of AmiA and AliA of streptococcal isolates compared to *S. pneumoniae* strain D39.

	AmiA		AliA	
	DNA (1980 bp)	Protein (659 aa)	DNA (1983 bp)	Protein (660 aa)
*S. pneumoniae* R6	99.95%	99.85% (1)	100%	100% (0)
*S. pneumoniae* ATCC 17619	99%	*	99.04%	99.39% (4)
*S. pseudopneumoniae* 410.05	97.93%	98.03% (13)	89.55%	92.88% (47)
*S. mitis* 3308.46	77%	81.64% (63)	80.5%	86.97% (86)

AmiA and AliA genes of bacteria used in the growth curves, *S. pneumoniae* strains R6 and ATCC 17619, clinical *S. pseudopneumoniae* isolate (410.05) and clinical *S. mitis* isolate (3308.46), were sequenced and translated. Sequence similarity was determined by BLAST to the respective D39 sequence over the whole length of 1980 bp (base pairs)/659 aa (amino acids) for AmiA and 1983 bp/660 aa for AliA. Total number of amino acids that differ between strains are indicated in brackets flowing the percent homology for the protein sequences. * stop codon producing truncation at 14 aa.

**Figure 6:**
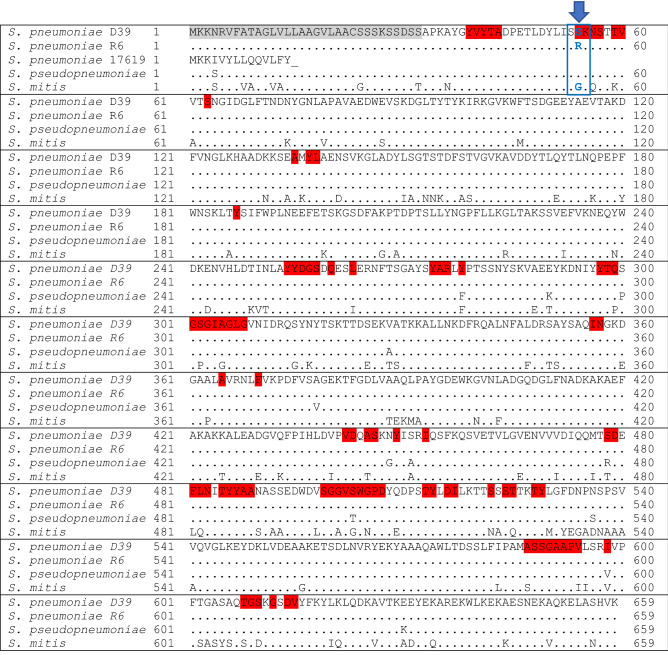
Protein sequence alignment of AmiA from *S. pneumoniae* strains D39, R6 and ATCC 17619; clinical *S. pseudopneumoniae* isolate (410.05) and clinical *S. mitis* isolate (3308.46). Amino acids highlighted in grey are part of the N-terminal signal peptide. Amino acids highlighted in red were predicted to be at the AmiA peptide ligand-AmiA protein interaction interface by PepNN, interaction propensity/probability of ≥ 0.5. Identical residues are represented by a dot(.). Translational stop codon represented by an underscore (_). Note that the only sequence difference between D39 and *S. pseudopneumoniae* (inhibited by AmiA peptide ligand) and R6 and *S. mitis* (not inhibited by AmiA peptide ligand) is at position 54 (indicated by blue arrow), predicted to be within the binding site.

We found that mutation in AmiA binding site or AmiA protein truncation correlate with loss of growth inhibition by AmiA peptide. Amino acids that were predicted to be at the peptide-protein interface of the AmiA peptide ligand and the AmiA protein (from Fig. 5a), are highlighted in red in the multiple sequence alignment in Fig. 6. In *S. pneumoniae* R6, the strain most closely related to *S. pneumoniae* D39, the only amino acid change for AmiA was detected at position 54: serine to arginine, supporting the prediction from Fig. 5a that its location is within the binding site. This amino acid was also changed in *S. mitis*. Growth of both *S. pneumoniae* strain R6 and *S. mitis* was unaffected by the AmiA peptide (Table 2). In *S. pneumoniae* strain ATCC 17619 we found one adenine missing compared to the eight consecutive adenines found near the beginning of *amiA* in strain D39. The resulting frameshift introduces a premature stop codon after fourteen amino acids (Fig. 6). Nevertheless, by mass spectrometry we did detect peptides matching the AmiA protein sequence downstream of the stop codon (Supplementary Table S1), albeit at an approximately 400-fold lower amount than we found in D39.

**Figure 7:**
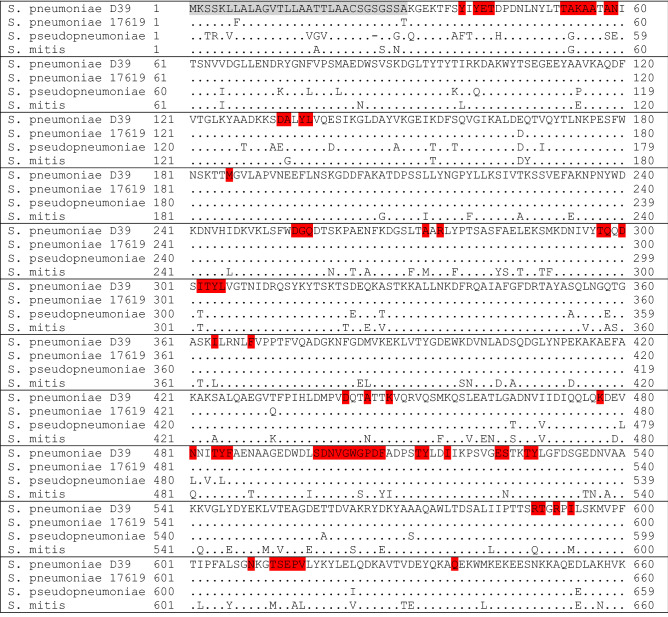
Protein sequence alignment of AliA from *S. pneumoniae* strains D39 and ATCC 17619; clinical *S. pseudopneumoniae* isolate (410.05) and clinical *S. mitis* isolate (3308.46). Amino acids highlighted in grey are part of the N-terminal signal peptide. Amino acids highlighted in red were predicted to be at the AliA peptide ligand-AliA protein interaction interface by PepNN, interaction propensity/probability of ≥ 0.5. Identical residues are represented by a dot(.). Gaps are represented by a minus (−).

We also detected mutations in AliA protein. Amino acids predicted to be at the peptide-protein interface of the AliA peptide ligand and the AliA protein, are highlighted in red in the multiple sequence alignment in Fig. 7. The sequence of *S. pneumoniae* strain R6 was not included in Fig. 7 due to 100% sequence identity to that of strain D39. In the AliA protein sequences, amino acid changes were detected outside and inside the predicted peptide-protein interaction interface. A few positions were mutated in both *S. pseudopneumoniae* and *S. mitis* and predicted to be involved in peptide binding, for example Ala53, Ile302 and Asn481. We note that at position 166 we found the same amino acid change from glutamic acid (E) to aspartic acid (D) for *S. pneumoniae* strain ATCC 17619, *S. pseudopneumoniae* and *S. mitis.* However, since the amino acid sequence of AliA of strain D39 (growth inhibited by AliA peptide ligand) and R6 (not inhibited by AliA peptide ligand) were identical, we compared the genomic sequences of strains D39 and R6 to look for difference outside of *aliA* which may explain the differences in response of the two strains to AliA peptide. We found a total of 88 genetic differences between D39 and R6, summarized in Supplementary Table S2, including missense variants of catabolite control protein A, *ccpA*, and transcriptional regulators *nusA* and *ybbH*. Details of all genomic differences between D39 and R6 strains are shown in Supplementary Table S3.

Our previous studies indicated that for strain 110.58, AmiA protein was able to bind AliA peptide as well as AmiA peptide^12,13^. We therefore tested the effect of AliA peptide on the growth of D39 and D39 AmiA knock out mutant (D39ΔAmiA). AliA peptide suppressed the growth of D39 but this effect was partially reversed in the D39ΔAmiA mutant (Supplementary Fig. S3). This is compatible with the binding partner for AliA peptide in D39 being AmiA protein. Therefore, the single amino acid change in R6 AmiA compared to D39 may explain the lack of effect of AliA peptide as well as AmiA peptide.

## Discussion

The substrate-binding proteins of pneumococcal Ami-AliA/AliB ABC transporter bind peptides with sequences found in ribosomal proteins of *Klebsiella pneumoniae*^12^. This results in changes in protein expression especially of those associated with metabolism and cell wall synthesis^13^. Here, we have shown that such peptides are released by *K. pneumoniae* during culture, compatible with our hypothesis that this is a novel route of interspecies bacterial communication. We also showed that co-culture with *K. pneumoniae* suppressed the growth of *S. pneumoniae*. Of the peptides tested, the optimal length resulting in suppression of pneumococcal growth appeared to be from nine to eleven amino acids. However, we have not excluded that shorter peptides may bind as previously proposed for peptides two to seven amino acids long^20^. A study on the peptide receptor of the Opp oligopeptide transport system (Ami-AliA/AliB homologue) of *Lactococcus lactis* suggests that small peptides are completely enclosed by the binding protein, whereas longer peptides might partially protrude from the binding pocket^21^. We hypothesize that the peptides which do not have the appropriate length cannot enter the binding pocket of the Ami-AliA/AliB oligopeptide permease failing to bind and therefore not inhibiting the growth of *S. pneumoniae*.

AmiA and AliA peptides suppressed the growth of pneumococci of diverse genetic backgrounds indicating that this system of detecting the microbial neighbourhood is likely to be widespread in the pneumococcal population. Sensing peptides derived from other bacteria and having steady but persistent growth might be an advantage in colonization in a hostile environment or when nutrient availability is limited^13^. The AmiA and AliA proteins are highly conserved between pneumococcal strains. This is encouraging for the potential use of such peptides as therapeutic agents against a broad range of pneumococcal strains. In contrast, the effect may be specific to pneumococci as the growth of other bacterial species did not appear to be affected, with the exception of AmiA peptide on *Streptococcus pseudopneumoniae*, a very close relative of *S. pneumoniae.* This highlights the potential of such peptides to avoid causing dysbiosis of the microbiota, in contrast to conventional antibiotics. In addition, both AmiA and AliA peptides were able to suppress the growth of antibiotic-resistant pneumococci, further pointing to their therapeutic potential in the treatment of disease where conventional antibiotics have failed. However, we found that the AmiA and AliA peptide ligands did not inhibit two *S. pneumoniae* strains tested: nonencapsulated strain R6 and serotype 19A ATCC 17619 strain. The fact that we found two pneumococcal strains whose growth was not suppressed by AmiA or AliA peptides indicates that it would be important to monitor development of any resistance to future peptide-based therapy. However, a comparison of the sequence of AmiA and AliA in streptococci which did and did not respond to the peptides, along with protein structure predictions, allowed us to gain insight into the location of the peptide-binding sites. So far, there is no experimentally validated structure of the AmiA or AliA proteins of *S. pneumoniae*. Our AlphaFold models of AmiA and AliA were of high confidence and enabled us to make peptide-protein binding predictions. Most peptides do not induce conformational changes on their partner upon binding and tend to bind in the largest pockets available on the protein surface^22^ and for OppA, peptides it binds are buried by the Venus flytrap mechanism^23^. The amino acids of AmiA and AliA that are predicted to be at the peptide-protein binding interface are located in what we hypothesize are the binding pockets. Specifically, position 54 is predicted to be at the AmiA peptide ligand-AmiA protein interaction interface, is located in the potential binding pocket and was the only amino acid difference between *S. pneumoniae* strain D39 (growth inhibited by AmiA peptide) and R6 (not inhibited). This amino acid difference at position 54 between strains D39 and R6 confirmed the finding of Lanie et al.^24^. Although we did not show conclusively that position 54 determines the difference in response to the AmiA peptide ligand since there are other SNPs in the genome, we note that this is the only amino acid difference between D39 and R6 in the AmiA sequence. A limitation of the in silico predictions is that we did not validate the protein structures and binding pocket of the ligands experimentally. However, the analysis revealed which amino acids are likely to be at the peptide-protein interaction interface. We hypothesize that the AmiA peptide ligand did not inhibit *S. pneumoniae* strain ATCC 17619 due to a frameshift resulting in a premature stop codon, which is partially ignored, resulting in a very low amount of AmiA protein. Insufficient AmiA protein means insufficient receptor for the peptide ligand and therefore no suppression of growth.

For AliA protein we could not identify a single amino acid change causing loss of binding although we did note amino acid changes in the AliA protein of *S. pneumoniae* ATCC 17619, *S. pseudopneumoniae* and *S. mitis* compared to D39. For *S. pseudopneumoniae* and *S. mitis* we found mutations inside and outside the predicted peptide-protein interaction interface but for *S. pneumoniae* strain ATCC 17619 we only found mutations in amino acids outside of the predicted peptide-protein interaction interface. The AliA sequences of *S. pneumoniae* strains D39 and R6 were identical even though AliA peptide inhibited growth of D39 but not R6. We speculated that there must be mutations in other parts of the R6 genome that affect the signaling of the AliA peptide ligand and the growth-inhibitory effect. Although identification of the mutation(s) responsible was beyond the scope of this study, comparison of the two genomes revealed a total of 88 genomic differences: We note with interest that one of these is a missense mutation in the catabolite control protein A gene, *ccpA*. CcpA is a master regulator of transcription affecting expression of up to 19% of the genome affecting particularly carbohydrate metabolism and therefore growth^25,26^. We have found previously that AliA peptide can bind AmiA protein of pneumococcal strain 110.58^12^. AliA protein of strains D39 and R6 are identical but AmiA protein differs by one amino acid in the predicted binding site, and AliA peptide suppressed growth of D39 but not R6. We therefore speculate that in strain D39 AliA peptide binds AmiA protein and not AliA protein.

In summary, our results are in accordance with our hypothesis that pneumococci sense the presence of *K. pneumoniae* via a novel peptide-mediated route of communication. We found that co-culture with *K. pneumoniae* suppressed the growth of *S. pneumoniae* and that peptides previously shown to bind to the substrate-binding proteins AmiA and AliA of the pneumococcal Ami-AliA/AliB permease were detected in the *K. pneumoniae* secretome. Peptide length affects function and for AmiA we have identified the location of an amino acid in the putative binding site whose mutation results in loss of response to the peptide. The effect of AmiA and AliA peptides on suppressing growth appears to be specific to pneumococci and widespread within this species, including antibiotic-resistant strains. We therefore propose their potential value as future therapeutic agents against pneumococcal diseases.

## Methods

### Bacterial strains and culture conditions

The bacterial strains were stored at − 80 °C in the Protect Microorganism Preservation System (Technical Service Consultants Ltd.). Swiss clinical *S. pneumoniae* strains for this study originated from a strain collection obtained from a nationwide surveillance for nasopharyngeal *S. pneumoniae* isolates from children with respiratory infection^27^. Clinical strains of *S. pneumoniae* of various genetic backgrounds were used (serotypes in brackets): 111.46 (19F), 202.58 (1), 208.41 (7F), 307.14 (18C). Pneumococcal reference strains ATCC 700903, ATCC 17619, D39 and R6 were also used^28,29^ and a D39 knock out mutant of AmiA (D39∆AmiA)^13,16^, as were reference strains of the other bacterial species: *H. influenzae ATCC 49247, S. aureus ATCC 29213, M. catarrhalis ATCC 8176* and *Klebsiella pneumoniae ATCC BAA-1706.* Swiss clinical isolates of commensal streptococci *S. pseudopneumoniae* (410.05) and *S. mitis* (3308.46) were sequenced and analysed as described previously and the sequence data submitted under the study accession number PRJEB2340 with the sample accession numbers SAMEA1022899 (410.05) and SAMEA1022891 (3308.46)^30^.

*S. pneumoniae* and *M. catarrhalis* were streaked out on CSBA plates and grown overnight at 37 °C and 5% CO_2_. *H. influenzae* was streaked out on CHOC plates and grown overnight at 37 °C and 5% CO_2_. *K. pneumoniae* and *S. aureus* were streaked out on CSBA plates and grown overnight at 37 °C and atmospheric CO_2_ concentration. All agar plates were made in-house.

To allow loading of a larger volume of culture medium for peptide extraction, we reduced the concentration of aromatic amino acids and some vitamins as suggested^31^ in the original CDM recipe^32^. Specifically, no folic acid was used and 100 µl of the biotin and riboflavin stock solution was added per litre of CDM. For *H. influenzae ATCC 49247* only, 20 ml of haemin chloride stock solution (concentration of haemin chloride: 500 µg/ml) was added per litre of CDM.

### Co-culture of *K. pneumoniae* and *S. pneumoniae*

*K. pneumoniae ATCC BAA-1706* and *S. pneumoniae* strain D39 were streaked out separately on CSBA plates and grown overnight at 37 °C and 5% CO_2_. Overnight cultures were prepared in BHI medium and cultured at 37 °C for approximately 4 h for *K. pneumoniae* and 7 h for *S. pneumoniae* until the cultures reached OD_600nm_ = 0.5, which corresponds to mid-log phase. 500 µl of the overnight culture was sub-cultured in 5 ml of fresh BHI until OD_600nm_ = 0.5, then centrifuged at 3000 g for 7 min and the pellet resuspended in 5 ml CDM. This was diluted in CDM to OD_600nm_ = 0.05 for *S. pneumoniae* and OD_600nm_ = 0.04 for *K. pneumoniae* to give approximately 1 × 10^7^ cfu/ml*.* Monocultures were prepared by combining 500 µl of CDM with 500 µl of bacterial suspension, co-cultures by combining 500 µl of the *K. pneumoniae* and 500 µl of the *S. pneumoniae* bacterial suspensions. The cultures were incubated in a water bath at 37 °C and dilutions streaked out on CSBA plates at the timepoint of inoculation, after 3 and 4.5 h. The CSBA plates were incubated at 37 °C and 5% CO_2_ and colonies counted the next day.

### Visualization of live/dead cells by microscopy

At exponential growth phase OD_600nm_ of 0.5, the analysis timepoint for peptide extraction, *K. pneumoniae* cells were stained using the LIVE/DEAD™ *Bac*Light™ assay kit (Thermo Fisher Scientific). Two aliquots of each 1 ml of bacteria were centrifuged at 10,000 g for 10 min. As a positive control for dead bacteria, one pellet was resuspended in 1 ml 70% ethanol and incubated for 1 h. The other pellet was resuspended in 1 ml 0.85% NaCl. The staining was performed as described in the manufacturer's protocol. 5 µl of stained bacteria suspension were pipetted onto a microscope slide and the coverslip added firmly. The slides were viewed using a Zeiss Axio Imager M1 fluorescence microscope with a 40 × objective. Pictures were taken with the Zeiss AxioCamHRc camera with merged fluorescent channels. To enumerate percent live bacteria, a total of 200 bacteria in 9 pictures were counted, classifying green as live bacteria and red as dead bacteria.

### Peptides

Unmodified synthetic peptides (Thermo Fisher Scientific) were used.

### Identification of peptides in the secretome

Peptides secreted by *K. pneumoniae* were analyzed as follows: Bacteria were grown in 100 ml of CDM until OD_600nm_ of 0.5, which corresponds to the exponential growth phase. Following centrifugation at 4000 g at 4 °C for 15 min, the supernatant was filtered through a 0.22 µm pore-size low-protein binding filter (Merck Millipore Ltd) and trifluoracetic acid added to a final concentration of 0.1%. Peptide isolation was performed by solid-phase extraction on Strata-X 33 µm Polymeric Reverse Phase cartridges with a vacuum manifold set (Phenomenex). The column was conditioned with 100% acetonitrile, equilibrated with water and 100 ml of sample loaded. Next, the column was washed with 10 ml of water. Increasing acetonitrile solutions from 10 to 100% were used for the elution steps. The eluted peptide fractions were merged and concentrated in a SpeedVac SPD131DDA concentrator and the sample stored at 4 °C until peptide identification by mass spectrometry. The data for three biological replicates was analyzed.

The samples were acidified to a concentration of 0.1% in formic acid. The peptides were sequenced on a QExactive HF mass spectrometer coupled with an Easy-nLC 1000 (Thermo Fisher) with one injection of 5 µl eluate. Peptides were trapped on a pre-column [C18 PepMap100, 5 m, 100 Å, 300 m × 5 mm (Thermo Fisher Scientific)] and separated by backflush on a C18 column (C18, 3 m, 155 Å, 0.075 mm i.d. × 150 mm length, Nikkyo Technos) by applying a 40 min gradient of 5% acetonitrile to 40% in water, 0.1% (V/V) formic acid, at a flow rate of 350 nl/min^−1^. Data acquisition was in data-dependent mode on the top 15 peaks with mass-to-charge ratio (m/z) between 360 and 1400, charge state 2–7, and no exclusion time set. Survey full scan MS resolution was set at 60,000 (at 400 m/z), automatic gain control (AGC) target of 1e6 with a maximum fill time of 50 ms. Higher energy collisional dissociation (HCD) fragmentation was carried out with a normalized collision energy of 28%, AGC of 1e5, maximum injection time of 110 ms, and spectra were recorded with a resolution of 15,000. Fragment spectra interpretation was done with PEAKS Studio Xpro using the following search parameters: parent and fragment error tolerances at 10 ppm and 0.01 Da, respectively, no protease enzyme specificity allowing C-terminal cleavage after any amino acids, a maximum of three dynamic amino acid modifications per peptide by oxidation on Met and deamidation on Asn/Gln deamidation on searching against all *K. pneumoniae* protein sequences present in the Uniprot-tremble database from February 2021. All peptides were filtered to a 1% FDR controlled by a concatenated target-decoy sequence search strategy (FDR on the PSM level = 0.6%, on protein group level = 4.1%).

### Identification of AmiA protein variant in *S. pneumoniae* strain ATCC 17619

Bacteria pellets were re-suspended in 50 µl lysis buffer (8 M Urea/100 mM Tris–HCl pH8, with Roche complete protease inhibitor cocktail) and sonicated with two short bursts of 5 s at 10 s intervals on ice. The protein concentration was measured by Qubit Protein Assay (Invitrogen). An aliquot of 10 µg protein was reduced, alkylated and digested with LysC for 2 h at 37 °C followed by trypsin at room temperature overnight^33^. The resulting peptides were separated by nano-liquid chromatography on an Ultimate 3000, (Thermo Fischer Scientific) and analyzed on an on-line coupled LUMOS tribrid orbitrap mass spectrometer (Thermo Fischer Scientific), with one injection of 500 ng peptides. Sample loading was done onto a pre-column (C18 PepMap 100, 5 µm, 100 A, 300 µm i.d. × 5 mm length) at a flow rate of 10 µl/min with 0.05% TFA in water/acetonitrile 98:2. After loading, peptides were eluted in back flush mode onto a self-packed C18 CSH Waters column (1.7 μm, 130 Å, 75 μm × 20 cm) with integrated emitter tip by applying a 90-min gradient of 5% acetonitrile to 40% in water/0.1% formic acid, at a flow rate of 250 nl/min. Data acquisition was made in data dependent mode with precursor ion scans recorded in the orbitrap at resolution of 120,000 (at m/z = 250) parallel to top speed fragment spectra of the most intense precursor ions in the linear trap with a cycle time of 3 s.

Fragment spectra interpretation was carried out with MSFragger^34^ against the most recent *S. pneumoniae* sequence database (version 2022_01) containing non-redundant sequences from taxonomy identifier 1313, and strains Hungary 19A and D39, respectively, concatenated with its reversed decoy sequences. Search parameters were as follows: precursor and fragment mass tolerances of 10 ppm and 0.4 Da, isotope error on first and second isotope, strict trypsin cleavage rule with a maximum of two missed cleavages per peptide, oxidation of Met and protein N-terminal acetylation as variable and fixed carbamidomethylation on Cys modifications. Peptide spectrum matches and protein identifications were filtered to a 1% false discovery rate using decoy matches.

Furthermore, the same cell digests were analyzed in data-independent acquisition mode (DIA) using the same instrumentation with fragment spectra acquisition in the orbitrap at resolution 30 k. AmiA peptide intensities based on the six most intense y-ion fragments were extracted from the DIA data using the spectrum library created with DDA with Skyline version 21.2.0.536. Protein abundance ratios between strains ATCC 17619 and D39 were calculated based on the sum of all peptide MS1 signals from DDA and averaged MS2 fragment signals from DIA data.

### Growth assay

Following overnight culture on agar plates, bacteria were sub-cultured in BHI medium overnight for approximately 7 hours until the bacteria cultures reached OD_600nm_ = 0.5, which corresponds to mid-log phase. 500 µl of the overnight culture was sub-cultured in 5 ml of fresh BHI until OD_600nm_ = 0.5, then centrifuged at 3000 g for 7 min and the pellet resuspended in 5 ml CDM. Growth was monitored in sterile flat-bottomed 96-well microtiter plates (Thermo Fisher Scientific) based on the method of Brewster^35^. To avoid condensation, the lid of the 96-well plates was pre-treated with 2 ml 0.05% Triton X-100 in 20% ethanol and air-dried before use. To 200 µl of CDM, 8 µl of bacteria was added. Concentrations of peptide ranged from 0 to 0.5 mg/ml. In a microplate reader (PerkinElmer and Tecan Trading AG), OD_450nm_ was measured at 37 °C every 30 min over 22 h with automatic shaking 5 s before each measurement. MIC_50_ was defined as the peptide concentration (mg/ml) that inhibited growth of the indicated strain by at least 50% compared to the control culture without peptide. This was adapted from Ovchinnikov et al.^36^. Growth curves were plotted in R with error bars representing SEM of 3 independent biological replicates.

### Bioinformatics

Genomic DNA was extracted from *S. pneumoniae* strains D39, R6 and ATCC 17619 with the QIAamp DNA Mini Kit (Qiagen) according to the manufacturer's protocol. High-throughput sequencing was performed using the Illumina Nextera DNA Flex assay (V2, 2 × 150 bp, 300 cycles) on an Illumina MiSeq benchtop sequencer (Illumina) according to the manufacturer’s protocols. Sequencing data was processed and assembled using the *shovill* pipeline (https://github.com/tseemann/shovill) with the –trim option and default parameters (v0.9.0). The Whole Genome Shotgun project has been deposited at DDBJ/ENA/GenBank under accessions JAMLJP000000000 (*S. pneumoniae* ATCC 17619), JAMLJQ000000000 (*S. pneumoniae* R6), and JAMLJR000000000 (*S. pneumoniae* D39). The assembled, non-annotated contig files were used in the BLAST with the D39 reference nucleotide sequences of *amiA* (locus_tag = SPD_1671) and *aliA* (locus_tag = SPD_0334). Next, the nucleotide sequence was found in the contig file and copied from start to stop codon and translated into the protein sequence with the Expasy Translate tool (https://web.expasy.org/translate/). Sequence similarity on protein level was determined with multiple sequence alignment with BLAST. The 3D protein structures with corresponding *.pdb files were modelled using the AlphaFold Colab notebook available at https://colab.research.google.com/github/sokrypton/ColabFold/blob/main/AlphaFold2.ipynb. The *.pdb files obtained together with the AmiA or AliA peptide ligand sequence were used to predict the peptide-protein interaction interface with PepNN-Struct. PepNN was installed and run as described in https://gitlab.com/oabdin/pepnn on UBELIX (http://www.id.unibe.ch/hpc), the HPC cluster at the University of Bern. The peptide-protein interaction interface was visualized with PyMol (The PyMol Molecular Graphics System, Version 2.5.2 Schrödinger, LLC).

### Ethics

All research was performed in accordance with the relevant guidelines and regulations.

## Supplementary Information

Supplementary Information 1.

Supplementary Information 2.

Supplementary Information 3.

Supplementary Information 4.

## Data Availability

All data generated or analyzed during this study are included in this published article. Whole Genome Shotgun project has been deposited at DDBJ/ENA/GenBank under accessions JAMLJP000000000 (*S. pneumoniae* ATCC 17619), JAMLJQ000000000 (*S. pneumoniae* R6), and JAMLJR000000000 (*S. pneumoniae* D39).
