# Supplementary material for: AmiA and AliA peptide ligands are secreted by Klebsiella pneumoniae and inhibit growth of Streptococcus pneumoniae
Source: Sci Rep. 2022 Dec 23;12:22268. doi: 10.1038/s41598-022-26838-z (PMC9789142; doi:10.1038/s41598-022-26838-z)
Supplement: Supplementary file 1 — Supplementary Information 1. [file 41598_2022_26838_MOESM1_ESM.docx]

**Supplementary Table S1 – Peptide sequences detected by mass spectrometry in *S. pneumoniae* strain ATCC 17619 matching to AmiA protein**

DDA 1^st^ rep

| **Peptide Sequence** | **Start** | **End** |
| --- | --- | --- |
| QALNFALDR | 341 | 349 |
| NLFVKPDFVSAGEK | 368 | 381 |
| NFTSGAYSYAR | 266 | 276 |
| AYGYVYTADPETLDYLISSK | 36 | 55 |
| ENVHLDTINLAYYDGSDQESLER | 243 | 265 |
| AVDDYTLQYTLNQPEPFWNSK | 164 | 184 |
| WFTSDGEEYAEVTAK | 105 | 119 |

DDA 2^nd^ rep

| **Peptide Sequence** | **Start** | **End** |
| --- | --- | --- |
| TYLGFDNPNSPSVVQVGLK | 528 | 546 |
| GSDFAKPTDPTSLLYNGPFLLK | 202 | 223 |
| NLFVKPDFVSAGEK | 368 | 381 |
| GLADYLSGTSTDFSTVGVK | 145 | 163 |
| AVDDYTLQYTLNQPEPFWNSK | 164 | 184 |

DIA

| **Peptide Sequence** | **Start** | **End** |
| --- | --- | --- |
| AVDDYTLQYTLNQPEPFWNSK | 164 | 184 |
| AYGYVYTADPETLDYLISSK | 36 | 55 |
| ENVHLDTINLAYYDGSDQESLER | 243 | 265 |
| NFTSGAYSYAR | 266 | 276 |
| NLFVKPDFVSAGEK | 368 | 381 |
| QALNFALDR | 341 | 349 |
| WFTSDGEEYAEVTAK | 105 | 119 |
| YAAAQAWLTDSSLFIPAMASSGAAPVLSR | 569 | 597 |
