# Supplementary material for: AmiA and AliA peptide ligands are secreted by Klebsiella pneumoniae and inhibit growth of Streptococcus pneumoniae
Source: Sci Rep. 2022 Dec 23;12:22268. doi: 10.1038/s41598-022-26838-z (PMC9789142; doi:10.1038/s41598-022-26838-z)
Supplement: Supplementary file 2 — Supplementary Information 2. [file 41598_2022_26838_MOESM2_ESM.docx]

**Supplementary Table S2 - Genomic differences between D39 (used as reference) and R6**

## (snippy analysis)

Summary

| **Variant Types** | **N=** | **Example** |
| --- | --- | --- |
| SNP  (Single Nucleotide P olymorphism) | 78 | A => T |
| COMPLEX  (Combination of SNP/MNP) | 4 | ATTC => GTTA |
| MNP  (Multiple Nucleotide Polymorphism) | 1 | GC => AA |
| DEL  (Deletion) | 4 | GC => G |
| INS  (Insertion) | 1 | G => GC |
| Total | 88 |  |

| **Functional effects of the SNPs** | **N=** |
| --- | --- |
| frameshift_variant | 1 |
| frameshift_variant&missense_variant | 2 |
| missense_variant | 54 |
| stop_gained | 2 |
| stop_lost&splice_region_variant | 1 |
| synonymous_variant | 15 |
| unknown | 13 |
| Total | 88 |
