# Supplementary material for: AmiA and AliA peptide ligands are secreted by Klebsiella pneumoniae and inhibit growth of Streptococcus pneumoniae
Source: Sci Rep. 2022 Dec 23;12:22268. doi: 10.1038/s41598-022-26838-z (PMC9789142; doi:10.1038/s41598-022-26838-z)
Supplement: Supplementary file 4 — Supplementary Information 4. [file 41598_2022_26838_MOESM4_ESM.docx]

**Supplementary Figures**

| **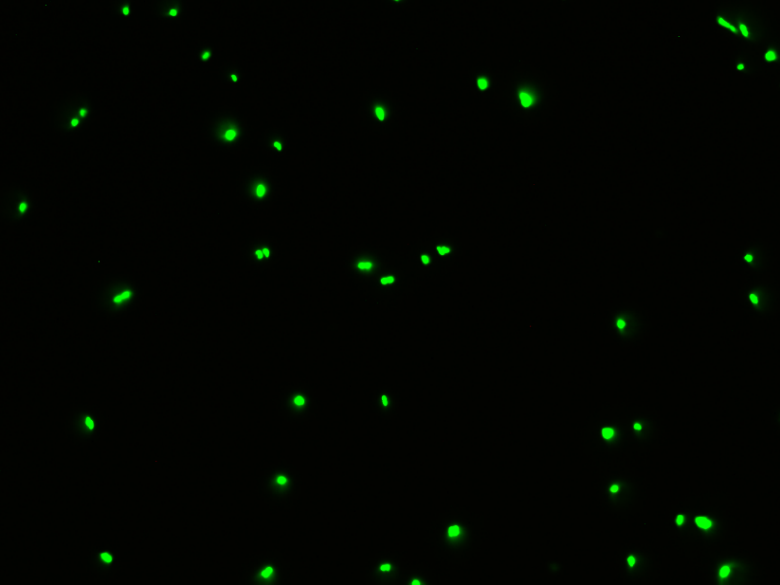a** | **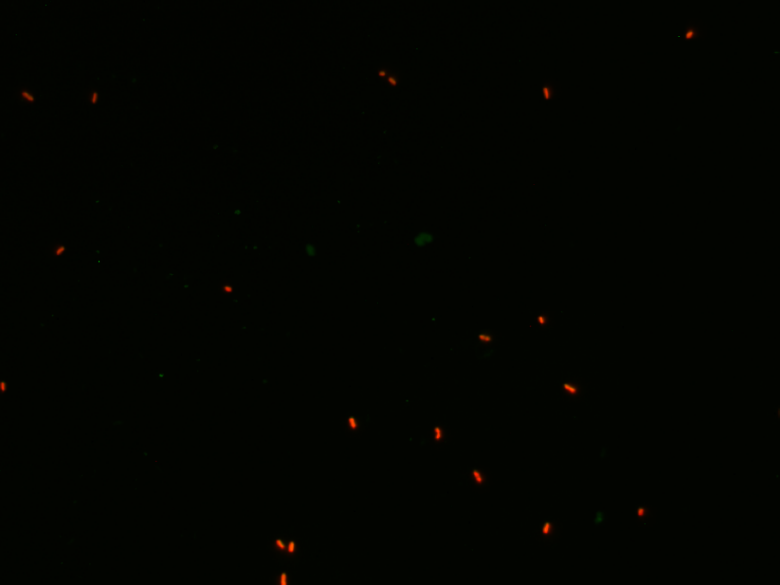b** |
| --- | --- |

**Supplementary Figure S1:** Results of LIVE/DEAD *Bac*Light bacterial viability assay visualized by fluorescence microscopy showing a) *K. pneumoniae* bacteria are alive (stained green) at exponential growth phase, equivalent to the growth phase at which the secretome was taken for peptide identification. b) Control showing dead *K. pneumoniae* (stained red) following treatment with 70% ethanol for 1 hour.

**
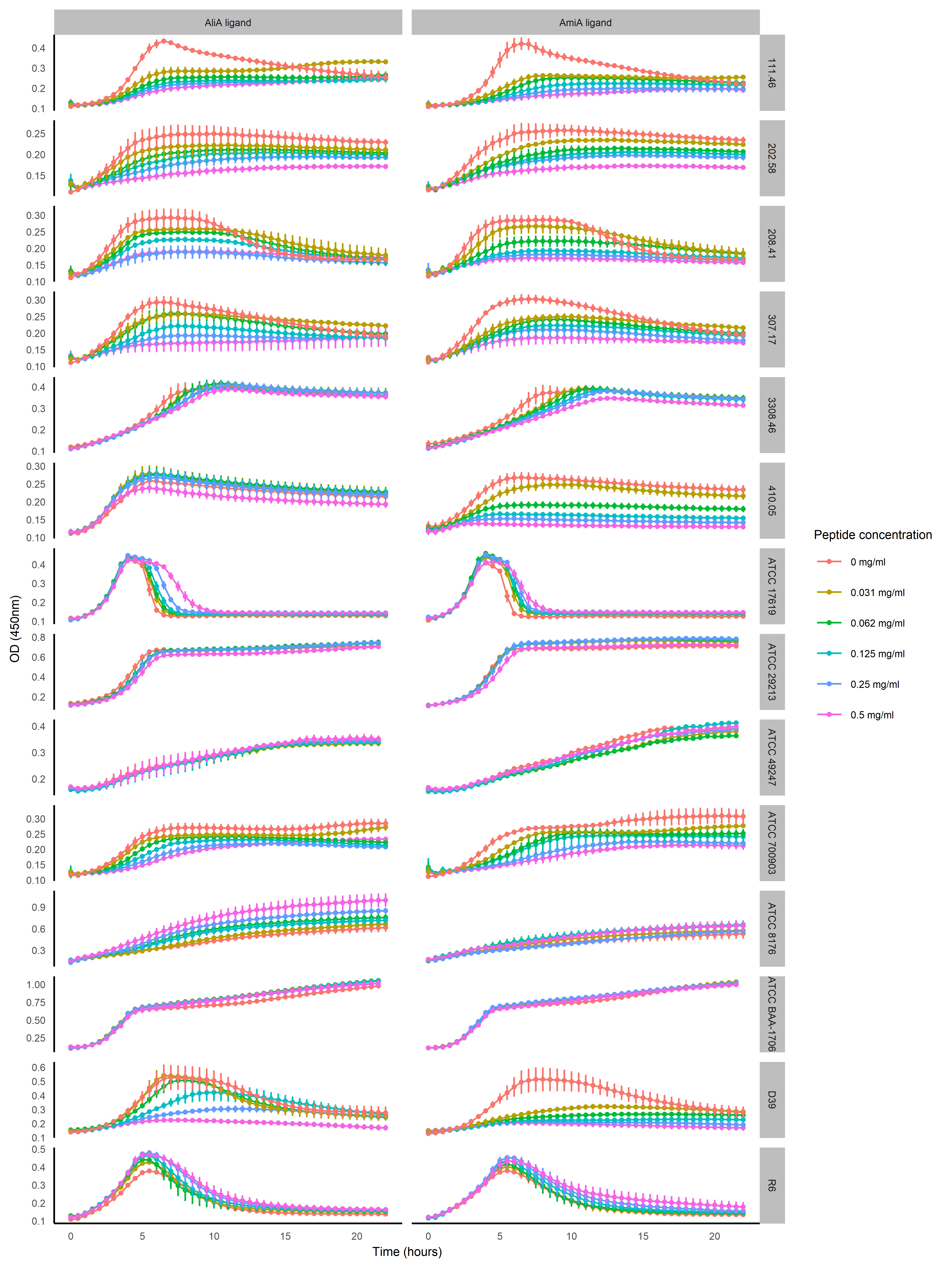
Supplementary Figure S2:** Growth curves of bacterial strains used in Table 2 for MIC_50_ calculation. Growth was measured in peptide-free chemically defined medium (CDM) in presence or absence of AmiA or AliA peptide ligand by measuring optical density (OD) over time. Results represent 3 independent experiments, error bars indicate SEM.

| 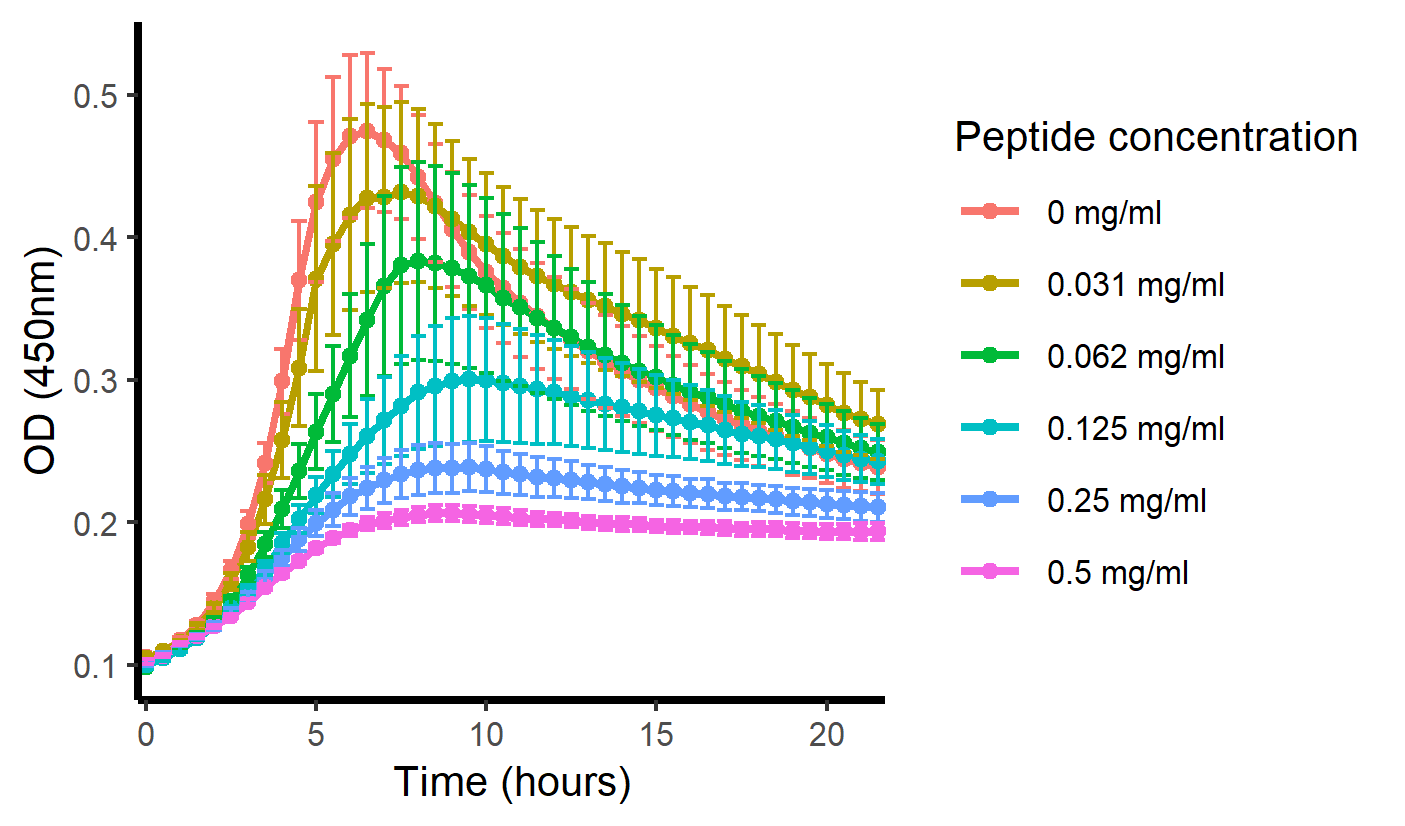 **a *S. pneumoniae* strain D39 with AliA peptide ligand** |
| --- |
| 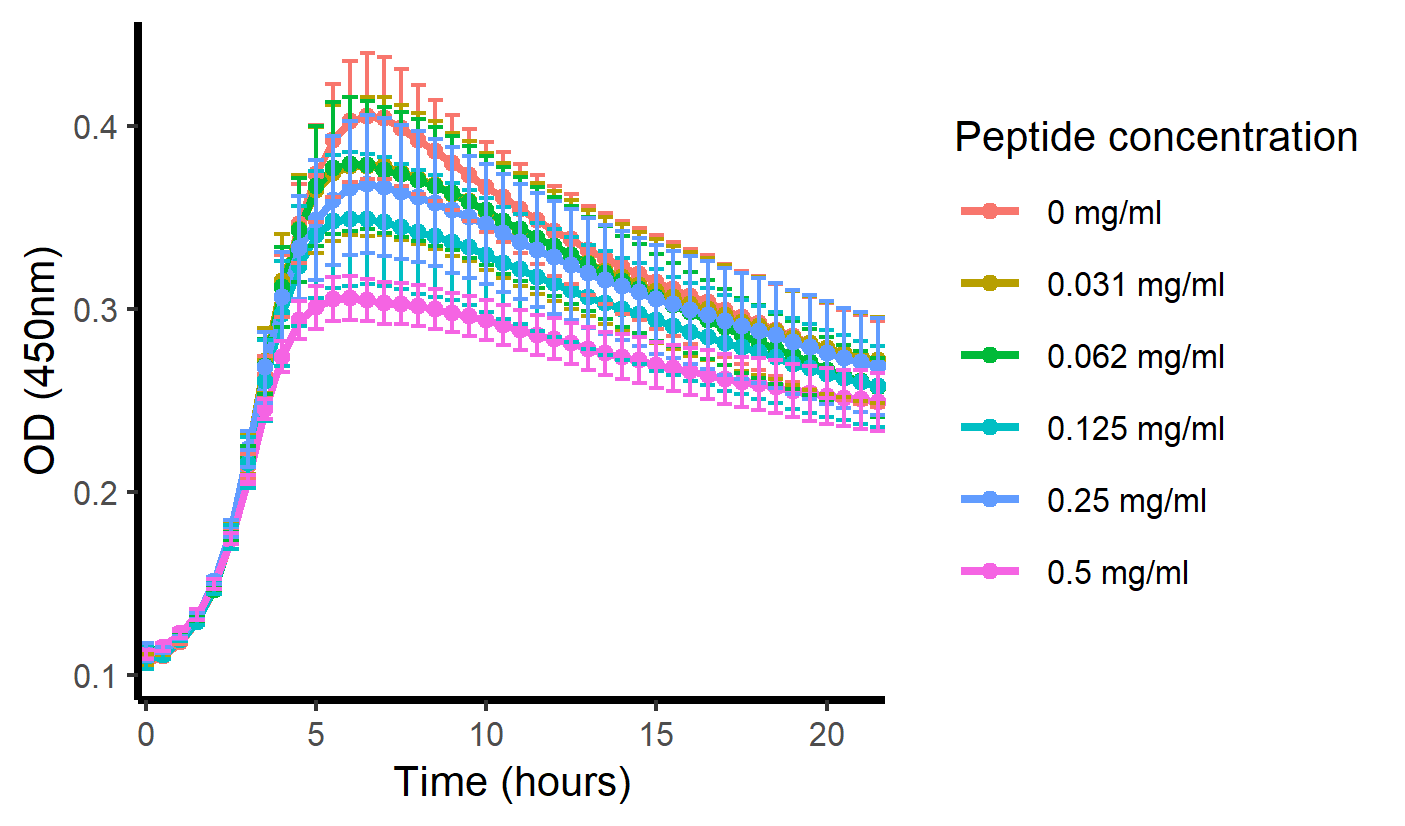**b *S. pneumoniae* strain D39 ∆AmiA with AliA peptide ligand** |
|  |

**Supplementary Figure S3:** Growth curves of *S. pneumoniae* strain D39 and its ∆AmiA mutant in the presence and absence of AliA peptide at the concentrations shown. (a) Growth of D39 is suppressed by AliA peptide but this effect is partially reversed in the D39∆AmiA mutant (b), compatible with the hypothesis that for strain D39 AliA peptide binds to AmiA protein.
